# Immunotherapy Benefit in a Patient With Non-Small Cell Lung Cancer and a Rare BRAF Mutation

**DOI:** 10.7759/cureus.11224

**Published:** 2020-10-28

**Authors:** Rebekah Rittberg, Shantanu Banerji, Susan Green, Gefei Qing, David E Dawe

**Affiliations:** 1 Oncology and Hematology, University of Manitoba, Winnipeg, CAN; 2 Oncology and Hematology, CancerCare Manitoba, University of Manitoba, Winnipeg, CAN; 3 Oncology and Hematology, CancerCare Manitoba, Winnipeg, CAN; 4 Pathology, University of Manitoba, Winnipeg, CAN

**Keywords:** immune checkpoint inhibitor, driver mutation, non-small cell lung cancer, braf, nivolumab

## Abstract

Immunotherapy is less effective in non-small cell lung cancer (NSCLC) with driver mutations in epidermal growth factor receptor *(EGFR)* or anaplastic lymphoma kinase *(ALK) *and some may extrapolate this trend to other driver mutations. Up to 4% of NSCLC cases contain a *BRAF* mutation. Most *BRAF* mutations are V600E, and little is known about the impact of treatment in rare *BRAF *G469A mutations. We present a case of a patient found to have *BRAF *G469A mutated NSCLC. She was diagnosed with Stage IIIB NSCLC and treated with concurrent chemotherapy and radiation. Post-treatment imaging demonstrated disease progression and she was started on nivolumab, resulting in a dramatic and prolonged response which is ongoing after 76 cycles. Her substantial response and prolonged benefit suggest that *BRAF*-mutated NSCLC may respond better than *EGFR*- or *ALK*-driven disease to immunotherapy. Due to the rarity of specific mutations, this case adds to the limited current published literature on NSCLC harbouring a *BRAF *G469A mutation and suggests that immunotherapy is a reasonable treatment option.

## Introduction

The therapeutic approach to non-small cell lung cancer (NSCLC) has dramatically shifted with identification of targetable driver mutations and the introduction of immune-checkpoint inhibitors (ICI) [1]. Treatment algorithms for tumors possessing epidermal growth factor receptor (*EGFR*) mutations and anaplastic lymphoma kinase (*ALK*) translocations involve targeted tyrosine kinase inhibitors then cytotoxic chemotherapy, before considering ICI monotherapy due to concerns about poor efficacy of ICI in the driver mutation-activated population [2]. Other activating mutations have been identified, including the *BRAF* gene, representing potential sites for direct acting or downstream therapeutic targets where the impact of ICI is not known [3].

## Case presentation

**Figure 1:**
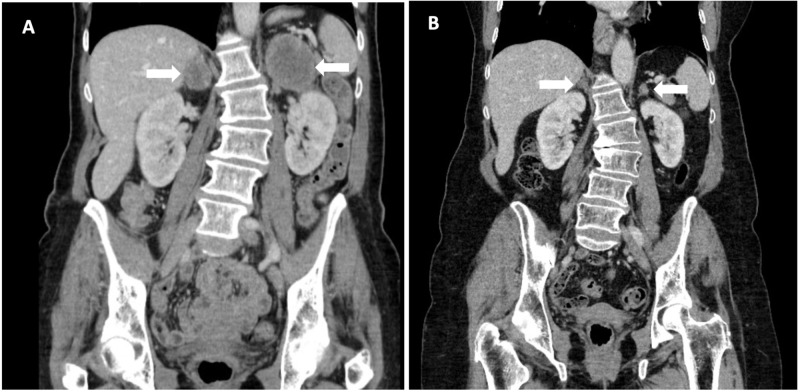
Treatment response with nivolumab A: CT abdomen before initiation of nivolumab demonstrating 6.0 x 4.4cm left adrenal mass and 3.1 x 2.5 cm right adrenal mass. B: Near complete resolution of bilateral adrenal masses after 41 months of nivolumab.

Here we present a case of a 61-year-old female, previous smoker, diagnosed with Stage IIIB NSCLC, adenocarcinoma subtype, programmed death-ligand 1 (PD-L1) >50%, *ALK* translocation, *EGFR* mutation, and *KRAS* mutation-negative and *de novo* *BRAF *G469A mutated. She was originally treated on a clinical trial with radical radiation (66 Gray in 33 fractions) and concurrent chemotherapy (cisplatin and etoposide), randomized to receive metformin in combination. Post-treatment computed tomography (CT) demonstrated improvement of her right hilar mass, but new 6.0 x 4.4 cm left adrenal mass and 3.1 x 2.5 cm right adrenal mass (Figure 1). She started nivolumab alone, with near-complete response after 33 cycles. She has received 76 cycles of nivolumab with ongoing disease control more than four years later. Her treatment has been complicated with Grade 1 diverticulitis and recurrent Grade 1 pneumonitis which has required steroid treatment.

## Discussion

Somatic activating *BRAF* mutations were first described in 2002 and are best recognised in melanoma, identified in up to 66% of patients [4,5]. *BRAF* mutations are also found in several other malignancies including 100% of classic hairy cell leukemia, 50% of papillary thyroid cancer, and 15% of colon cancers [4,6]. NSCLC genomic studies have found that 1-4% of all NSCLC, predominantly adenocarcinoma, harbor a *BRAF* mutation and have similar survival as patients with wild type *BRAF* [7-10]. *ALK-*positive and *EGFR* mutant NSCLC are found more frequently in non-smokers and within the Asian population, while *BRAF* mutations appear to be more common in current or past smokers and the Caucasian population [3,7,8,10]. Over half of NSCLC *BRAF* mutations are V600E (exon 15), the remainder being non-V600E (exon 11 or 15). G469A accounts for approximately 23% of NSCLC *BRAF* mutations, however very little is published on patients whose cancer carries this mutation [10].

Dual *BRAF* inhibition with dabrafenib and vemurafenib significantly improves overall survival in advanced melanoma [5]. In *BRAF* V600E mutant metastatic NSCLC, an objective response rate (ORR) of 33-42% is seen in response to the same treatment, without a similar response observed in non-V600E *BRAF* carriers [11-13]. Dual *BRAF* and mitogen-activating protein kinase pathway inhibition, using a combination of dabrafenib and trametinib, respectively, has also been employed in *BRAF* V600E mutant metastatic NSCLC and found to have a 61-63% ORR. Unfortunately, *BRAF* non-V600E mutations were excluded from the trial [14]. With these results the Food and Drug Administration (FDA) approved dabrafenib plus trametinib for *BRAF* V600E mutant metastatic NSCLC [15]. Although these treatment options are promising, they cannot be applied to patients with *BRAF* G469A mutations. ICI therapies have now proven to be an effective option for many cancers [16]. However, only a portion of patients respond and experience the benefits of long-term disease control. In patients with NSCLC, the likelihood of response can be partially predicted by the level of PD-L1 expression on cancer cells, but this biomarker has proven far less useful in other malignancies [17,18].

*EGFR*- or *ALK*-mutation-positive NSCLCs typically have shorter and less complete response to ICI, compared to NSCLC without these oncogenic drivers, even with high PD-L1 expression [2]. This reduction in benefit results in ICI monotherapy being left until late in the treatment algorithm for NSCLC with *EGFR-* or *ALK-*driven NSCLC and some would extrapolate this concern to other driver mutations. Multiple hypotheses on this diminished response have been proposed, including lower tumor mutation burden or tumor-infiltrating lymphocytes, microenvironment differences, and an alternate escape pathway that is not associated with PD-1/PD-L1 [17]. Most current ICI clinical trials exclude patients with *EGFR*-mutant- or *ALK-*positive NSCLCs and most new data is achieved through small subgroup analysis and real-world data. Whether *BRAF*-mutant NSCLCs also have a similar response to ICI remains unclear. Guisier et al. recently considered the efficacy of ICI in Stage IV NSCLC harboring *BRAF, HER2, MET* mutation, or *RET* translocation. Twenty-six patients with *BRAF* V600E mutations demonstrated a 26% ORR with a median overall survival of 22.5 months. Comparatively, 18 patients had *BRAF* non-V600 mutations demonstrating a 35% ORR and 12.0 months median overall survival with ICI, but the specific G469A subgroup was not reported. Patients were predominantly treated with nivolumab; PD-L1 expression was ≥50% in 11% of patients and unknown for 56% [9]. Our *BRAF *G469A mutant NSCLC index case experienced a deep and durable response. Given that *BRAF* mutant NSCLC is found more commonly in current or past smokers, and is associated with high tumor mutation burden, strong response to ICI might be reasonable to expect [1].

## Conclusions

Research is evolving in the evaluation of rare oncogenic drivers. Due to the rarity of specific mutations, accrual of patients in clinical trials is challenging. This profound and prolonged response to an ICI adds to the limited current literature on NSCLC harbouring a *BRAF* G469A mutation and further suggests that ICI may be of benefit and a reasonable treatment option.
